# Two Cases of Concussion of the Brain*Read before Central of Georgia Railway Surgeons’ Association at Macon, April 20, 1897.

**Published:** 1897-09

**Authors:** M. A. Clark

**Affiliations:** Macon, Ga.


### TWO CASES OF CONCUSSION OF THE BRAIN.*

By M. A. CLARK, A.M., M.D.,

MACON, GA.

Beeman A., colored, aged eleven; family history good; previous health good. He was playing with a companion on the depot wharf. Finding an empty whisky barrel, they decided to try the effects of a lighted match placed in the bung. The result was a violent explosion; a boy blown several feet into the air, and falling, striking his head against a telegraph pole. I was called immediately, and found the following: Patient lying on back; stertorous breathing; deep stupor; pulse 160, very weak and compressible; temperature 96 F. I expected to find the skull compressing the brain, but found only a slight contused wound of the scalp in the parieto-occipital region.

I had patient moved to my office; warm applications to body, and gave 1-60 grain strychnine nitrate hypodermically, to stimulate the flagging heart. I dressed the contusion antiseptically, and, placing patient in a comfortable position, watched results. I repeated the strychnia in about an hour, the heart having become much depressed again. In about two hours patient vomited a large quantity of watermelon. Still unconscious, paying no heed to even loud calling. As early as his condition would allow I had him carried home and put to bed. I gave him hydrarg. chlor. mit. gr. v. and sodii bicarbonate gr. x., and ordered ℥i. magnesii sulphate on the

*Read before Central of Georgia Railway Surgeons' Association at Macon, April 20, 1897.

morrow. I ordered cold cloths put to head, perfect quiet and liquid diet.

On the following morning his pulse was normal, temperature normal, fully conscious, and he was begging for watermelon. Continued cold to head; instructed perfect quiet and liquid diet. The next morning the patient was able to sit up, and wanted to get up and go outdoors to play with other children. I dismissed him, with instructions for him to be kept quiet for a few days. He has never shown any ill effects from the injury.

*Case 2.*—August 3, Miss Roberta H., white, aged nineteen, visiting from New Orleans, La., her aunt in Barnesville; family history good; previous health good. She was out bicycle riding with two friends. While going down a small hill at the rate of about ten miles per hour, her rear wheel struck a brickbat, and the tire being torn asunder, was thrown from under her. She fell on the hard road, striking solidly on her buttocks. She was taken to the nearest house, and I was called. Being engaged, my brother Dr. B. J. Clark answered the call and found the following condition: Patient unconscious; screaming occasionally, and holding her hand to her head; pupils irregularly contracted, but responding to light; pulse rapid and feeble; temperature 96 F.; respiration shallow and jerky. Could elicit no reply to any questions; very nervous and restless. Gave gr. 1-100 hyoscine hydrobromate to quiet, and put hot applications to body and extremities.

I saw her in about one hour, and found her in deep stupor; respiration shallow and rapid; pulse 140; temperature 96 F.; no signs of consciousness. The heart being very feeble, we gave gr. 1-30 strychnine nitrate, and gr. 1-100 nitroglycerine hypodermically. She rested quietly all night.

August 4, 8 a.m. Stupor pronounced; on loud calling she would open her eyes and stare vacantly, but make no reply; pulse 120; temperature 97 F.; breathing deeper and more nearly regular; sighs frequently. Gave hydrarg. chlor. mit. gr. vi., sodii bicarbonate gr. xx., to be followed at one o'clock in the afternoon by ℥i. magnesii sul., and put cold cloths to head.

1:30 p.m. Complains of her head; makes no effort to understand anything. Her only reply to all questions is, "Oh, my head."

4 p.m. Temperature 98 degrees; pulse 100; respiration about normal; still complans of head; answers no questions; stupor well marked.

August 5, 8 a.m. Had a fairly good night. Complains of head. When asked if there is any other pain replies: "Oh, my head!" Cannot see. Speaks only when loudly called and violently shaken, then lapses into stupor. Temperature 101 degrees; pulse 110. Gave ℥ j. magnesii sulphat. to move bowels. Gave potassium and sodium bromide gr. xv., each every three hours. Put ice pack to head.

2 p.m. No special change. Continued same treatment.

8 p.m. Very restless; complains more of head; temperature 101½; face much flushed. Continued ice to head, and gave gr. 1-100 hyoscine hydrobromate to quiet.

August 6, 8 a.m. Rested well and looks better; complains more of head and inability to discern objects; irritable when disturbed; wishes to remain quiet all the time; takes nourishment only when urged, and then very small quantities; temperature 100 degrees; pulse 100; respiration normal. Ordered copious enema to move bowels. Continued ice to head, and gave the bromide solution every two hours. Applied mustard to nape of neck.

2 p.m. Temperature 101; pulse 108; complains of head, and then lapses into deep sleep; still unable to see; notices no one, and never speaks save to say, "Oh, my head." Continued treatment as above.

August 7, 8:30 a.m. Temperature 99.6; pulse 90; seems more nearly rational; recognizes her aunt's voice for the first time; still unable to distinguish objects, and complains of head. Gave the bromides every four hours, and alternated with salophen gr. x. Continued ice to head.

4 p.m. Complains more of head; evidently more sensitive to pain; more sensitive to light; begs to be allowed to sleep; temperature 100; pulse 108; took a little gruel with apparent relish.

August 8, 8:30 a.m. Very restless during night, sleeping but little; temperature 99 degrees; pulse 100; begs to be permitted to sleep. Left off bromides, and gave salophen gr. x. every three hours.

3 p.m. Pulse 100; temperature 99.6 degrees; seems much improved; able to discern objects in part; recalls having fallen from wheel but remembers nothing more; complains of head and of being so tired; begs to go to sleep. Gave at 8:30 trional gr. x. to make sleep.

August 9, 8:30 a.m. Slept well during night; says for the first time that she is better; temperature 98.6; pulse 84; still complains of head; sits up in bed, but prefers to lie quietly and begs to be not disturbed; takes more nourishment and seems to relish it. Gave potassium and sodium bromid. gr. xx. every four hours. Continued cold to head.

August 10, 9 a.m. Much improved, though still complains of head; slight difficulty of vision; temperature 98.4 degrees; pulse 80. Left off bromides; put on general tonics, and continued cold applications to head.

August 11-16. Improvement very marked. Able to sit up on the 16th, and was free from all pain. Improved rapidly for the next several days, and was able to return to her home in New Orleans on the 23d; making the trip without any ill effects whatever.

The points of interest are these: One was caused by direct violence to head; the other by indirect violence. Shock was apparently as profound in one case as the other, though the former recovered much more readily. The one from indirect violence resulted in a genuine encephalitis and had to be treated as such, making a slow recovery. The freedom from headache in the one struck on the head, and the severity and persistence of headache in the other, although head was not struck.

I use the term concussion here merely because it is the one generally employed, and not because I prefer, or even like the term. Like Keen, White, our President, and others, I think laceration a more appropriate term, and wish that concussion were omitted entirely from our text.

